# Democratic health in the corona pandemic. The corona pandemic as a trigger or amplifier of democratic erosion or autocratization?

**DOI:** 10.1007/s12286-023-00558-8

**Published:** 2023-03-29

**Authors:** Marianne Kneuer, Stefan Wurster

**Affiliations:** 1 Technische Universität Dresden, Bergstraße 53, 01069 Dresden, Germany; 2 Technische Universität München, Arcisstr. 21, 80333 München, Germany

## Introduction

COVID-19 constituted an unprecedented “transboundary mega crisis” (Boin et al. 2020) that referred to all spheres of the states and societies. Originated in China and then spreading rapidly throughout the world it was probably the first truly global crisis generating an external shock for every country (Wurster 2021). On the political level, governments were confronted with a huge effort of crisis management domestically as well as in terms of regional and global coordination. On the economic level severe impacts on the overall production and thus on the well-being of economies stood at risk, provoking large-scale attempts of economic containment (Hörisch et al. 2022). While the public health systems were especially challenged, also on the societal level, enormous social cohesion and solidarity were needed and thus invoked by governments in order to tackle the crisis.

Over the course of 2022, a general perception has solidified among the public in many countries that the pandemic is “over”. This may also be due to the fact that many governments (with significant exceptions such as in China until end of 2022) have relaxed the remaining, albeit mild, measures such as mandatory masking and mandatory testing upon entry into a country (Oxford Coronavirus Government Response Tracker—OxCGRT n.y). At the same time, some experts consider the pandemic to have transformed to an endemic state, while there is actually no consensus within the health experts. In addition, the Russian invasion to Ukraine which undoubtedly and equally presents a crisis with global implications shifted the attention of governments at the national and global levels. Nevertheless, the impact of the pandemic remains a topic that is far from being fully explored, as the long-term consequences, in particular, of crisis management have not yet been fully analyzed. This also applies to the effects on the health of the democracy taking into consideration that it was a relatively long period, during which government policy was strongly influenced by the fight against the pandemic and considerable restrictions were imposed.

As unprecedented as the COVID-19 pandemic was, the measures governments took to counter it followed suit. Hence, in the wake of this Corona crisis, all governments were called upon to take mitigating measures, most of which represent far-reaching interventions in public life such as curfews, restrictions for domestic movements, international travel restrictions including entry restrictions, keeping distance and mandatory mask wearing, school closures, and closure of businesses (Hale et al. 2020; WHO). In fact, this menu of restrictions was practically in effect globally and reached a high diffusion within the international community in the first months of the pandemic between February 2020 and April 2020, the so called first wave. However, they did not only extend to the first few months of the pandemic, but in some cases have continued to this day (as of January 2023).

It is important to note that we can divide the pandemic into several phases (see Wurster 2021). In the first (initial) phase of the pandemic (from December 2019 until February 2020) noticing the local outbreak of the virus allowing individual cases to be tracked and creating general awareness of the problem was central. After failing to prevent a global outbreak of the Corona pandemic, the second phase focused on containing the exponential spread of the virus (“flatten the curve”) with all the robust (relatively undifferentiated) means available at the time. The government responses were comprehensive and aimed at protecting the public health systems and in consequence, human lives. At the same time, these measures implied significant imitations for the personal lives of citizens and their political rights: rules for public and private gatherings were adapted and thus the right to gather and to protest was limited, religious services were broadly suspended which meant restricting the right for exercising religion, schools were closed and thus the right for education limited, and finally, the stay-home-rules and all movement restrictions domestically and internationally interfered massively in the right of free movement. While these measures can be considered as vital to prevent the spread of infectious disease and mitigate its potential consequences, the limitations of democratic rights were prone to raise concerns of scholars and pundits if and to what extent democracy would also be quarantined, placed in lockdown or get infected by authoritarian measures and practices. Three scenarios enter in these considerations: Firstly, the unprecedented health emergency provides an opportunity for autocrats to instrumentalized the pandemic for upgrading repression and control. Second, the pandemic equally serves as an amplifier for illiberal incumbents to accelerate and intensify democratic erosion processes that are already under way. Third, even in democracies where governments have no intention to exploit the pandemic for non-democratic purposes, democratic quality decreases, mainly because executive action dominated weakening the legislative in face of dominance of executive action (Marschall 2020; Merkel 2020; Bolleyer and Salát 2021).

While the second phase (form March 2020 until approximately June/July 2020) was characterized by a high degree of “clarity” choosing far-reaching containment measures—despite the uncertainty about the exact nature of the virus—in most countries, handling the pandemic entered a third phase in summer 2020. In this third pandemic phase (in many countries from August 2020 until end of 2021), the complexity of political decision-making increased tremendously, since it came along with a re-differentiation of the goals. In addition to preventing a next wave of infection, the economic and social consequences of the crisis had to be addressed. This put additional pressure on the political decision makers, which had to balance out—more than in the phase before—different economic and social (“flatten the consequences of the pandemic”) as well as medical interests (Wurster 2021). Due to the successful development of effective corona vaccines many (western) countries entered in the course of 2022 a fourth phase of the now expiring pandemic. Based on successful vaccination campaigns, large parts of the population (herd immunity) and vulnerable groups were now better protected against severe disease progression, so that we saw in many countries a reduction of mandatory corona measures during the second half of the year 2022. Besides the handling of this, governments now also face momentum (window of opportunity) to answer the question, what might be the long-term lessons and consequences out of the crisis (fifth phase, in many countries since the second half of the year 2022). How to build preventive and resilient health, social, economic systems that are better prepared for a new pandemic than the old ones? For democracies, the question how to optimize democratic processes in order to react even faster and more effectively on crises like the Corona pandemic became also vivalent.

Within the course of the global pandemic situation, the issue of democratic health has gained research interest. However, there is a need for thorough and differentiated analyses on how the crisis management impacted democratic structures, processes and principles in different countries and regimes. Which areas have been affected by restrictions on democracy or have shown themselves to be particularly vulnerable to the use of emergency measures to undermine democratic standards? Have these restrictions been implemented formally and on the basis on democratic procedures or not? And how have the restrictions been justified? What role did the (lack of) success in fighting the pandemic play? These are only few still open questions.

Another democracy-related issue refreshed an already existing debate on the comparison of performance between democracies and autocracies. Thus, especially when the pandemic emerged the question raised whether restrictive mitigation measures would attribute an advantage to autocratic states and societies. An intensified competition which has existed even before the COVID-19 pandemic outbreak between the Western-style, liberal-law democracies on the one hand and emerging autocratically ruled countries (China, Russia, Singapore, etc.) on the other hand increased the pressure on democracies. The race between the political regimes for interpretive sovereignty has been continued and even reinforced in the context of the pandemic. Thus, Cheibub et al. (2020) find that on average, democratic governments were slower to react to the pandemic than autocratic ones (at least in the second phase of the pandemic, see below). Another essential question however was if democracies or autocracies did better in preventing deaths and how they handled the crisis when looking at its (broader) economic and social impacts. As we will elaborate further below, the picture here is rather mixed. Results vary when looking at the different five phases of the pandemic, or when elaborating its consequences for the economic, social or health sector. Finally, one can find indication that (systematic) differences within the world of democratic states as well as between different autocracies exist (Schmidt 2022). Without neglecting that regime type played a role in the handling of this pandemic other factors (e.g. experience with pandemics in the past) were important too.

This special issue offers a broad picture of the implications COVID-19 has; it presents analyses from three regions: Europe, Asia, and Africa. The decision to publish such a special issue with the temporal distance of almost three years has turned out to be advantageous. The time period makes it possible to transcend early and sometimes premature conclusions that are drawn from a short-term perspective—such as regarding only the first wave and the acute phases of the pandemic. This temporal distance allows us to evaluate the medium- and long-term performance of different crisis management strategies. It also helps us understand why some governments quickly reversed restrictive measures while others did not and assess the extent to which “pandemic backsliding” (V-Dem n.d.) may be establishing itself as a medium-term pattern.

## Covid-19 as a trigger for democratic erosion and authoritarian consolidation?

Severe crises bear critical features for those who are confronted with the crisis management, especially political leaders. First of all, crisis defined as critical junctures where a social system experiences an “urgent threat to its basic structures and fundamental values, which harbors many ‘unknowns’ and appears to require a far-reaching response” (Boin et al. 2017: 5). Besides this threat having the potential of disrupting lifelines and daily routines, secondly, crises are characterized by urgency insofar solutions have to be formulated as soon as possible and rapid action is demanded. Finally, crises imply a high degree of uncertainty, which also refers to the policy responses that are decided in reaction to the crisis (Boin et al. 2017: 6f). In the moment of responding to an identified crisis, it is not clear at all if the policy response is adequate and efficient. Hence, during the Covid-19 crisis the temporal dimension also played an important role: on one hand rapid action was required in the acute phase of the pandemic with high numbers of infected and high rates of mortality, on the other hand, governments then had to decide how to continue handling mitigating measures that went along with severe limitations to democratic rights. Therefore, it is of upmost importance to study the impact of crisis management on the quality of democracy during the pandemic in a longitudinal perspective embarking more than only the beginning and acute phase of the first months.

It is conventional wisdom that due to these characteristics and affordances, crises constitute the “hour of the executive”. This can imply that the legislative is not involved sufficiently, that rule of law is weakened as actors of horizontal or vertical accountability are not heard or consulted in the usual way, and even the opposition might reduce its ambition for controlling government in a general climate of ‘rallying around the flag’, following an approach of maintaining national cohesion when facing a dramatic threat. The pandemic indeed has opened quite a few such gateways for executive rule-through and circumvention of legislative procedures or other checks and balances in the political system. The mere fact that several states declared a state of emergency, which generally implies that executive powers are extended, and other procedures of democratic decision-making can apply, is a strong indication. Moreover, concerns were raised that the pandemic poses severe risks to the guarantee of genuine and transparent elections, and that elections could be cancelled, postponed or problems could arise with postal voting or electronic voting (Landman and Splendore 2020, p. 1061).

Considering the aforementioned temporal dimension, the insights of not few studies suggest, however, that there is more ambivalence in the governments’ reactions than anticipated. The findings indicate that (1) democratic governments to a great degree reversed restrictive measures regarding the democratic dimension relatively swiftly, and (2) that the pandemic did not foster the emergence of authoritarian elements, but accentuated already existing dysfunctionalities or struggles. At the same time, however, what becomes apparent is (3) that indeed regime types can make a difference, but again with varying results.

## Potentially affected dimensions of democracy

**Fig. 1:**
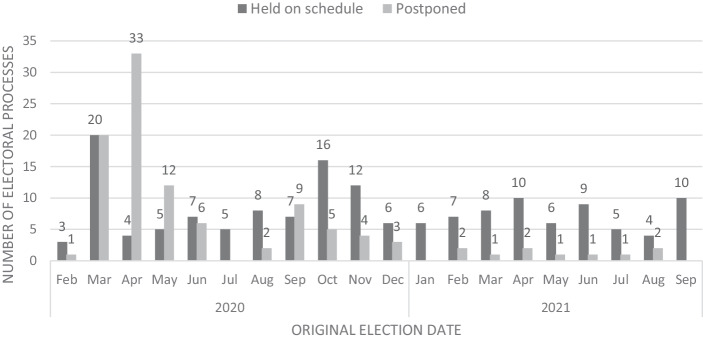
Elections held and postponed during Covid-19 (2/2020-9/2021). (Source: IDEA 2022)

In the following, we look at important democratic dimension: elections, democratic procedures and democratic liberties. If we look at *elections*, as a first point and taking into account that elections constitute a core function in democracies, the concern of severe disruptions of elections did not materialize. The intergovernmental Institute of Democracy and Electoral Assistance (IDEA), which monitors elections worldwide and gives a well-documented overview over the electoral practices during the pandemic, shows that between February 2020 and February 2022 80 elections were postponed, but at the same time the double were held as scheduled (IDEA 2022). More telling is the development over time: While postponement happened in March, April, May and June to a significant degree, in April being postponement the dominant way compared to only four elections that took place as scheduled, this trend clearly decreased in the following months until October 2021 (see Fig. 1).

Regarding election postponement regime type seems to play a role. James and Asplund show with IDEA data, that democracies are quicker to subsequently hold elections once delayed. Thus in 2020 (01.01.2020–31.08.2020), no democracies postponed without agreeing upon a new election date. However, there were worrying cases of one hybrid regime (Ethiopia) and two autocracies (Chad and Somalia), that have postponed but not rescheduled (James and Asplund 2020).

Furthermore, what became apparent during the pandemic were several vulnerabilities in the management of elections. It was however not Covid-19 that caused these vulnerabilities, rather “the pandemic laid bare pre-existing systemic limitations and inadequacies of established policies and practices” (Spinelli 2021). The effect of the pandemic was to reveal these problems in countries where they have been long time overlooked. While, it can even be concluded that the pandemic had a positive result in paving the way to substantial and necessary change of electoral management (Spinelli 2021), it still has to be observed what of these advancements will endure.

Besides elections, second, *infringements of democratic procedures* were widespread within both democracies and autocracies in 2020, and more so in autocracies. The Pandemic Backsliding Index (PanDem) created by the Varieties of Democracy Project measured authoritarian practices (emergency measures without time limit, disproportionate limitations on the role of legislatures, government disinformation campaigns) and illiberal practices (discriminatory measures, derogation of non-derogable rights, abusive enforcement) between March and December 2020 for 144 countries (Edgell et al. 2021). They found that around 80% of all countries violated democracy mainly on the basis of authoritarian practices and here it was predominantly the lack of limits on emergency measures, while less than 30% of the countries used illiberal practices. By far most violations (more than 70%) referred to restrictions on media, out of which more than 60% were assessed as major violations. Thus, media freedom was by far the most vulnerable area. According the PanDem most of these violations of accountability occurred in autocracies, but also some democracies were affected (Edgell et al. 2021). Looking at the development of violations, the trend shows that countries after the first acute phases of the pandemic 38% of the countries reversed restrictive measures. PanDem offers a good overview on the pandemic months of 2020, but the results have to be taken with some caution, as the short times series allow only to draw partial conclusion, as the authors themselves underline (Edgell et al. 2021, p. 6). So far, more comprehensive data covering 2021 are lacking.

However, it is evident with regard to emergency measures and the resulting violations of democratic procedures that equally here the time dimension played a role, insofar as the highly restrictive government responses were successively withdrawn. Legal shortcomings of first responses were mostly corrected later, e.g. by legal amendments where the legal basis had been unclear (Meyer-Resende 2020). In addition, there were protests by citizens or criticism by parliamentarians in a number of countries, already in 2020, but particularly in 2021, to which governments responded. Members of the German Bundestag, for example, demanded more say and more opportunities for debate on the measures.

Finally, looking at *freedom rights and civil liberties*, the restrictions have been massive, especially in 2020, but also beyond. The Oxford Coronavirus Government Response Tracker (OxCGRT n.y.) offers a complete longitudinal perspective from the beginning of the pandemic until today (OxCGRT n.y.). Regarding the cancellation of public events and public gatherings, the global picture reveals that from April 2020 on and until May 2022 (public gatherings) and October 2022 (public events) those countries were in clear minority which did not issue any measures while the rest of the world recommended or required the cancellation. The most severe measure has been the curfews depriving people from leaving their homes whereas other limitations referred to restrictions of internal movement or international travelling. Stay-home restrictions dominated (as requirement or recommendation) from April 2020 to April 2022 and stay-home requirements even until April 2021 in most of the countries. Restrictions on internal movement only were used a few months (until October 2020) as a measure by most countries, while international travelling in the majority of countries worldwide has been subject to different kinds of restrictions until October 2022.

In all, when assessing the impact of COVID-19 on democracy, several aspects have to be taken into consideration: First, the pandemic hit the global community in the midst of a longer evolution of democratic regression (Diamond 2021; Lührmann and Lindberg 2019). Thus, illiberal political leaders, democratic erosion and autocratization have been on the agenda in recent years, as democratic institutions and principles have been under attack. Not few scholars, therefore expected an additional backlash of democracy on a global scale when governments started to react to the pandemic. More than two years after the start of the pandemic, though, assessments suggest that the pandemic did not lead to further autocratization in 2020 (Hellmeier et al. 2021; V‑Dem Report 2021) and had a limited effect on the general and global downward trend of democracy in 2021 (V-Dem Report 2022). Although we see further autocratization, it seems that is not the pandemic that accounts for this global trend. However, as several studies suggest, the pandemic intensified already existing democratic deficits as Guasti shows in Eastern Central Europe (2020) or intensifies already existing struggles between democratic and anti-democratic forces (Youngs and Panchulidze 2020, p. 21). Therefore, it will be important to continue observing long-term effects on democratic quality, especially in those areas where goverments massively inferred such as media freedom. Concerns about long-term effects are not limited to institutional aspects. How governments managed communication and information during COVID influenced the openness, rationality, and quality of public debate. Many have used the pandemic as pretext to limit the public sphere). Thus, what long-term effect will manipulation of information and discrediting of scientific knowledge have?

Second, a strong concentration of decision-making power on the executive can be observed in most regimes, in democracies as well as autocracies. However, there is not a ‘one size fits all’ pattern (Kneuer 2022). Hence, as a benchmark for evaluating the mode and intensity of stretching executive competences, the criteria of “proportionate, necessary and nondiscriminatory” reflecting international human rights law are useful (UN Experts 2020; UN 2020). Here it is of special interest, if governments set time limits of emergency measures and if they limit the role of legislatures in a disproportionate way. This turned out to be the essential distinctive feature between liberal-minded democracies and those that use the favor of the hour for executive aggrandizement.

Third, democracies have not been completely immune against the violation of democratic rights, procedures and principles, but the more they have been committed to protect democracy before the pandemic, the more they were committed to uphold democracy during the pandemic. Thus, the degree to which democratic principles are protected during ‘normal times’ explain the governments’ willingness to constrain the freedom of individuals and to limit checks and balances during crisis times. The strength of the democratic institutions seems to influence the way how democracies handle the democratic tradeoff between protecting democratic principles and protecting health (Engler et al. 2021).

Forth, the least surprising outcome refers to autocracies and the assumed increase of repression and consolidation of autocratic regimes. The most compelling issue, though, is how democratic eroding and autocratizing governments evolved during the pandemic and how much they could benefit from the state of emergency produced by the pandemic. In this regard, the insight from the literature on democratic erosion informs about the intentionality of the dismantling of democratic institutions, rights, and principles. Scholars agree in that incumbents play the decisive role in implementing this process of democratic erosion (Bermeo 2016; Levitsky and Ziblatt 2018; Kneuer 2021). Inasmuch such dismantling is purposefully driven, this let assume that the same political leader would consider the pandemic an opportunity for further steps of executive aggradizement and justifying it with the crisis situation. Hungary is a case for that (see Schweiger 2023).

Fifth, a lot of studies that emerged covering the acute phase of 2020 only can display a partial view on the issue. The above undertaken tour d’horizon on the development of measures makes evident that the temporal dimension plays an essential role.

On one side we need more regime-sensitive comparison focusing on the variance of violations in the different regime types. On the other hand, the question of the performance of different regimes in the pandemic (Cheibub et al. 2020) is of crucial importance.

## Who performed better? The race between the regimes

Successful management of the pandemic became a key component for regime legitimation in the last years both for democracies and for autocracies. In the ongoing race for supremacy and interpretative sovereignty (Wurster 2022), regimes tried to proclaim success in this regard. For both political models, one can find arguments in the literature for being better equipped to navigate through the disruptive circumstances of a pandemic (see Merkel 2020; Schmidt 2022; Wurster 2022). Democracies try to legitimize themselves by satisfying the interests of a majority of today living citizens (Bueno de Mesquita et al. 2002; Wurster 2022). By various control mechanism (regular elections free public debates), democratic governments should be forced to act in the interest of current majorities. The ability of a democratic government to act is however restricted by different means (“checks and balances”, rule of law, protection of minorities, public discourse and guaranteed political participation rights for citizens). While decision-making processes in democracies became often slow and onerous (Cheibub et al. 2020), which can pose a serious thread in an acute crisis situation, a wide range of participation opportunities for different groups can also promote innovative problem solving and increase the ability of the system to correct errors and learn out of them (de Tocqueville 1840). Autocracies, on the other hand, boast that they give their governments every possible tool, so that they can act without significant resistance or restrictions. Especially in an acute crisis like a pandemic, this allows to concentrate all essential resources in achieving one prioritized goal (flatten the curve of infections) and force it through with brutal means (Siewert et al. 2020). Contradicting goals are pushed aside and taken at a back seat. The corresponding ability of autocracies to use a wide range of repressive measures is however accompanied with several problems. In an atmosphere of fear and forced government support, no one really dares to report negative news to the political leadership. This leads to an insufficient feedback loop hindering autocratic leaders to take problem-adopted decisions (Wurster 2013, 2022). The lack of government control in autocracies comes with another specific problem. It makes it more difficult to leave once chosen problem-solving paths that have proven to be disadvantageous, especially if the autocratic leadership interprets a reversal as an admission of weakness that could jeopardize their rule.

Both democracies and autocracies have specific characteristics that can prove to be either advantageous or disadvantageous in the different phases of the pandemic. They might also influence, how and to what degree a country prioritizes (conflicting) policy goals, during and in the aftermath of a crisis event.

For the first phase the COVID-19 outbreak, one can state a devastating failure of autocratic crisis management. Two problematic features of autocratic rule (insufficient feedback loop and concern of the autocratic leadership to admit a serious problem) played together in a disastrous manner, preventing effective containment in autocratic China, eventually triggering the global pandemic (Schmidt 2022). While local cadres’ feared to reported the local outbreak of the virus in Wuhan with the necessary urgency to the autocratic leadership, leading members of the Communist Party of China were primarily concerned in the first weeks after the outbreak to intimidate and lock away doctors and journalists who have warned of the virus, aiming at covering up the outbreak (Burkle 2020).

After this disastrous effect of autocratic steering in the first phase of the COVID-19 outbreak, autocracies like China or Singapore, backed with sufficient resources and a high level of state capacity, were able to exploit on of their core competencies in the second phase of the pandemic. They were able to force true one priority—in this case the reduction of COVID-19 infections and deaths—whatever it takes, while implementing ruthless instruments (mass testing, close-meshed contact tracing, strict confinements and rigorous lockdowns), curtailing individual liberties unthinkable in democracies (Tisdall 2020; Wurster et al. 2022). Accompanied by expanding medical and hospital capacities in record time (Merkel 2020) as well as implementing a high volume testing system (documented i.e. by high values at the Covid-19 Stringency Index, Mathieu et al. 2020; https://ourworldindata.org/covid-stringency-index), they were able to contain the virus in remarkably short time keeping infection numbers low, which also reflects relative low mortality rates in the course of the following years.

**Fig. 2:**
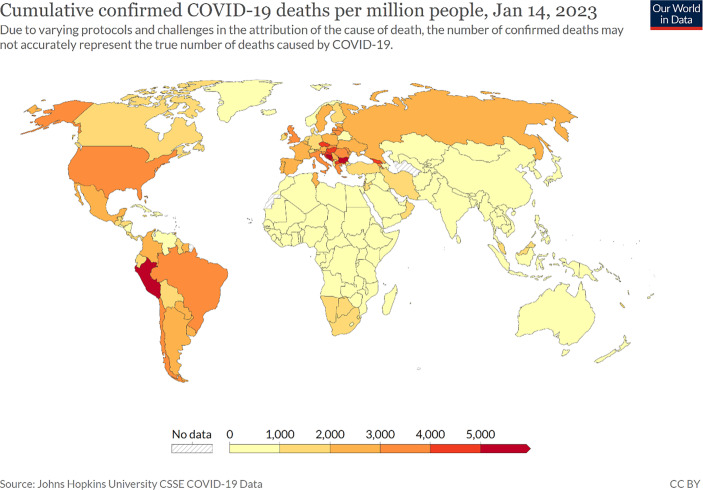
Cumulative confirmed COVID-19 death per million people worldwide. (Source: Mathieu et al. 2020; https://ourworldindata.org/covid-deaths. Accessed 15 January 2023)

As Fig. 2 shows, the cumulative confirmed COVID-19 deaths per million people vary tremendously between the countries after almost three years of the pandemic.

In doing so, we cannot find a clear democracy advantage regarding this key performance indicator for handling the pandemic. While there are some democracies, particularly in East Asia (Japan, South Korea, Taiwan, New Zeeland or Australia), that have managed to keep the COVID-19 death numbers relatively low, several western democracies (USA, Italy, Spain or France) rank among the most affected states. Even so not all autocracies (e.g. Iran or Russia) were successful, and there are serious doubts as to whether autocracies like China, which stopped transparently documenting its infection and death numbers at the end of 2022, Russia, Venezuela and many countries in Africa could or wanted to publish valid COVID-19 death numbers, we saw specific problems in some of the most developed western democracies to cope with an at least partial collapse of their health care systems and to prevent high death rates (Merkel 2020; Schmidt 2022), particularly in the first and second year of the pandemic. Besides the hurdles steering through incisive measures to fight the pandemic, the relatively high vulnerability of their aging societies, their close economic integration, which facilitates the spread of the virus, as well as the expanded diagnostic capacities when recording COVID-19 deaths (problem of unreported COVID-19 deaths in autocracies due to cover-up and incompetence), play an important role to explain these sobering findings for western democracies (Schmidt 2022).

**Fig. 3:**
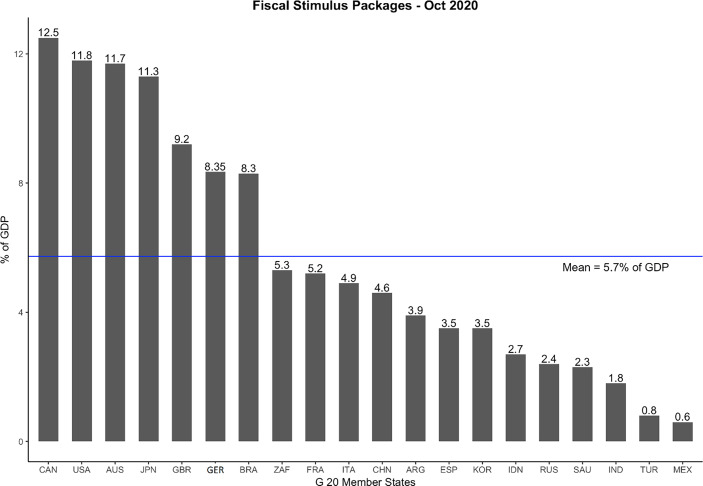
Size of Fiscal Stimulus Packages of the G20 states 2020 (in % of GDP). (Source: Based on Hörisch et al. 2022: 4)

The triumph of some autocracies outperforming democracies in the second phase of the pandemic when (only) looking at the infection and mortality rate, doesn’t however hold the longer the pandemic lasts and the more one widens the focus of the evaluation. Even so, autocracies like China managed to keep the infection numbers in their own country relatively low this came with a high price of increasing economic and social costs. To handle its widespread economic and social consequences by balancing out different goals became more and more important in the third phase of the pandemic. Open participation processes put pressure on democratic governments to address a broader segment of public interests and find innovative ways to “return to normality” without provoking a relapse into a next wave of infections (Wurster 2022). With regard to the economic consequences of the pandemic, both democracies and autocracies were hit hard by the pandemic, facing serious decline of GDP (in some countries of more than 20%) in 2020 (see Mathieu et al. 2020; https://ourworldindata.org/covid-health-economy). While autocratic China continued to focus its effort on a hard repression strategy until December 2022 carrying out tremendous cost for its economy (see Holbig 2023), a couple of democracies started already early in 2020 to address the economic consequences of the pandemic. Looking inter alia at the size of the fiscal stimulus packages of G20 countries in 2020 (see Fig. 3) conducted as reactions tackling the economic COVID-19 shock we find a leading group of countries exclusively consisting of established democracies (Canada, United States, Australia, Japan, Great Britain and Germany). Contrary to that, all autocracies among the G20 countries (China, Russia and Saudi Arabia) spend significantly less than G20 average to address the economic consequences of the pandemic. Even so one takes into account that most rich countries are democracies having an easier job to set up large fiscal packages, this finding remains remarkable.

**Fig. 4:**
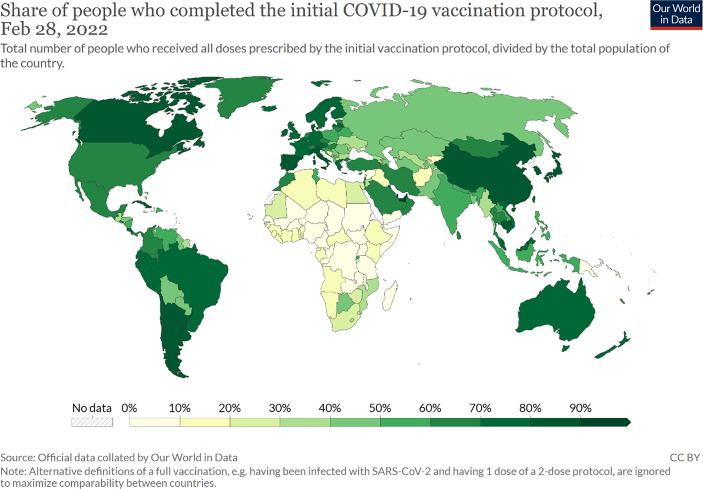
COVID-19 vaccination rate 2022. (Source: Mathieu et al. 2020; https://ourworldindata.org/covid-vaccinations. Accessed 15 January 2023)

Rapid advances in development of new mRNA vaccines, achieved in democratically governed countries of North America and Europe, finally opened the door for a fourth phase of an now expiring pandemic. As a game changer for “living with the virus”, a high vaccination rate made it possible to withdraw most of the corona-related restrictions, bringing back livelihood for millions of peoples even under the conditions of ongoing infection waves (Omicron and other variants). Even so, some countries faced organizational problems at the beginning of their vaccination campaigns by early 2022 most of western democracies achieved a high vaccination rate outperforming many of their autocratic competitors (see Fig. 4). Even in autocracies achieving a high vaccination rate, like in China, the population often faces a significant immunity gap since domestic vaccines had been less effective than the mRNA vaccines developed in the United States and Europe (Holbig 2023).

Under these conditions, western democracies get leeway to relax their COVID-19 regime, while autocracies like China where caught in a dead-end unable to adapt to the new situation. On the one hand, they were not willing to use effective vaccines from abroad, and on the other hand, they could not relax their strict COVID-19 regime. The “collective psychological trauma” (Cai 2022) caused by Communist Party of Chinas ideology seeing Zero-COVID as proof of the socialist system’s superiority over Western liberal democracy eventually caused in December 2022 the first serious nationwide protest in China (so called “White Paper Protests”) since the Tiananmen Square protests of 1989 and forced the Chinese government to drop the Zero-COVID strategy altogether in a dramatic way.

In its fifth phase, finally the pandemic converts into an endemic status. While doing so its structural long-term consequences become more and more important. What lessons states have learned out of it for similar crises in the future? Even so long-term planning is one of the Achilles heels of democratic governments that have to be very concerned about satisfying the current interests of today’s voters (Wurster 2013), the crisis opened especially for democracies a “window of opportunity”. It facilitated to break up encrusted structures and path dependencies stabilized by powerful distribution coalitions in democracies (Olson 1982). While non-responsive power structures in autocracies lead to an inflexible COVID-19 crisis management, strong public competition in democracies opened the room for learning processes and incentivised transformations towards more resilient systems better prepared to address future crisis. Concerning the ongoing competition between democratic and autocratic regimes, this could prove to be an important asset for democracies, considering escalating economic, social and environmental crisis in the coming decades (Schreurs and Wurster 2019).

## The contributions of the special issue

In this special issue, we have gathered five articles dealing with the issue of democratic health and/or the performance of democracies and autocracies during the pandemic in different regions around the world. While the first three contributions focus on countries in Central and Central Eastern Europa, one contribution offer a comparative perspective on different countries in Asia, and finally one article refers to South Africa. Seven countries are examined in more detail in the articles, including industrialized (Germany, Austria, South Korea and Singapore) as well as emerging countries (China, Hungary and South Africa). In addition to considerations on established democracies, the articles in the special issue also address hybrid regimes and autocratic states. In doing so, the special issue forms a broad basis for dealing with questions of democratic health and performance in democracies and autocracies from a comparative perspective.

In the first contribution by* Alexia Katsanidou, Marianne Kneuer, Felix Bensmann, Dimitar Dimitrov *and* Stefan Dietze *with the title: “Limitations of democratic rights during the Covid-19 pandemic—exploring the citizens’ perception and discussions on dangers to democracy in Germany” the authors deal with the question, whether people in Germany perceived a threat to democracy during the pandemic. Even though citizens of an established democracy, the population in Germany was confronted with severe limitations of democratic freedoms and rights during the pandemic. What effect did this had on the satisfaction rate of German citizens with the way democracy worked in Germany during the pandemic? Covering the period from December 2020 to August 2021 by using two independent sources of data (longitudinal panel survey and large-scale analysis of Twitter tweets), the authors are able to track the perceptions about democracy in Germany over time, and link citizens’ perceptions with their socio-economic status, political attitudes, and trust in government. As a key result the authors detect time relatedness (the longer the pandemic endured, the more concern on democratic damage increased) as well as the intensity of the pandemic (increasing COVID-19 cases and deaths) as relevant factors for the fluctuation of satisfaction with democracy during the pandemic in Germany (Katsanidou et al. 2023). They can also show that pandemic concerns for democracy were higher around May 2021, in comparison to December 2020, but then calmed again in August 2021. While the discourse on Twitter became more polarized in the course of the pandemic, clear patterns crystallyze for distinct differences among age and educational groups, as well as supporters of different parties. Especially less educated people, the age group between 40 and 60 and supporters of AfD perceived a threat for a damage of democracy during the pandemic while for the other groups such concerns were significantly lower.

*Maike Rump* and *Nadine Zwiener-Collins *equally are interested in the micro level of citizens’ attitudes during the pandemic, though with a different focus. They look at one of the measures limiting democratic rights, the restrictions on protests, and how the norm fulfillment of others affect one’s own democracy satisfaction. Thus, the authors do not examine the effect of government performance on democracy satisfaction, as some other studies have done, but they do address a relationship that has been rather little studied so far, namely the effect of others’ behavior. The assumption is that the perception of this protest influences how citizens evaluate the pandemic management and that this perception affects their satisfaction with democracy. The authors examine this correlation using Austria as an example. They are able to show that political trust, government satisfaction, the perceived health risk, trust in the health care sector, and social cohesion most strongly influence satisfaction with democracy.

Different to the first two contributions*, Christian Schweiger’s *article on: “Governance under the Covid-19 Pandemic: Comparative perspectives on Germany and Hungary” addresses the institutional level examining if and to what degree the pandemic influenced the democratic quality. Basing on two different cases—Germany as a liberal democracy and Hungary as a flawed democracy—the author is interested if the pandemic has accelerated the erosion process a fragile democracies such as Hungary and if it had adverse effects on functioning democracies. Scheiger shows that the German polity has not witnessed a fundamental shift towards executive powers at the expense of legislative and independent judicial supervision while in Hungary the Fidesz government under the leadership of prime minister Orbán has used the pandemic as a tool to at least temporarily remove existing constraints on its already substantial executive powers by instilling a new regime of governing by decree during times of crises. The author problematizes that this widening of the gap between the liberal democracies in the EU and backsliding democracies poses even bigger challenges to the EU itself, especially because the recent Ukraine crisis shows that Orbán is willing to instrumentalise any crisis for his political agenda.

Switching from Europe to Asia the next contribution of the special issue by *Heike Holbig* with the title: “Navigating the Dual Dilemma between Lives, Rights and Livelihoods: COVID-19 Responses in China, Singapore and South Korea” compares the pandemic response strategies of three Asian countries with each other. While on can consider China, Singapore and South Korea as success cases when it comes to the reduction of COVID-19 cases and mortality rate (especially compared to some western democracies like the United States or Italy) we see a clear variance between the three countries when balancing out the dilemma between the protection of lives, individual rights, and livelihoods during the pandemic (Holbig 2023). Also comparing three different regime types—autocratic China, hybrid Singapore and democratic South Korea—over the period from late 2019 to mid-2022 with each other *Holbig* finds remarkable differences in the adaptability of COVID-19 response strategies between the three countries. While China committed itself at the beginning of the second phase of the pandemic to a relentless zero-tolerance policy (rigorous lockdowns, economic insulation and nationalist entrenchment) and trapped into the path dependency of exclusively prioritizing the protection of lives while neglecting other goals, Singapore, which initially also opted for a zero-tolerance strategy, changed its approach over the course of the pandemic. Other than South Korea that never opted for “Zero-Covid” but modified its pandemic response all along, while finding a relatively smooth transition to a “Living with COVID” strategy, Singapore had to change its broader COVID-19 plan several times. The coordinated and inclusive strategy of democratic South Korea allowed a balanced achievement of “Lives, Rights and Livelihoods” during Corona, while Singapore had to learn a painful lesson of not neglecting certain groups in society, when COVID-19 spread among migrant workers in March and April 2020, forcing the government to introduce a partial lockdown. Contrary to the flexibility of South Koreas corona policy *Holbig* can show that the autocratic one party regime in China was stuck in a—at the beginning very successful—Zero-COVID strategy that became more and more dysfunctional by causing tremendous social and economic side effects and cost.

In the last contribution of the special issue *Dirk Kotzé* explores in his article with the title: “Public health and democratic governance: COVID-19’s impact on South Africa’s democracy” the impact of the pandemic management on South Africa’s democracy. South Africa provides an interesting case because of two reasons: on one hand side, the governance of the country served as an example for other southern African countries during the pandemic and thus has a wider regional influence. On the other hand, the constitution offers two paths for the pandemic management, one with more inclusion of and control by the parliament, and one with a strong and almost completely exclusive executive mandate. Due to the fact that the South African president Ramaphosa choose the latter path, several problems with democratic principles occurred. *Kotzé* shows that the limitations on the South African parliamentary processes during the pandemic definitely reduced the quality of South Africa’s democracy, both in terms of institutional autonomy (i.e., the separation of powers), oversight over the executive and decision-making. At the same time, however, all executive actions with pandemic emergency functions were concluded in 2022. Not least because of the negative public response, the executive role during the pandemic could not be entrenched and therefore it did not have a lasting effect on South Africa’s democracy. Thus, *Kotzé* concludes that while the quality of democracy has been negatively affected by the government’s pandemic management, the consolidated state of the country’s democracy is not under threat.

## Some tentative conclusions and futures research desiderata

The pandemic posed a double challenge for democracies. On the one hand, it was a question of overcoming the health, social and economic consequences of the pandemic as successful as possible in a competitive race against its autocracies counterparts. At the same time, it was also about maintaining the stability and vitality of democratic institutions and processes, even under the difficult conditions of a pandemic.

Regarding the most pressing question if and to what extent democracies—full-fledged or flawed—or hybrid regimes would use the pandemic to expand executive power and move towards authoritarianism, the contributions corroborate two important insights: First, democracies have shown a considerable resilience and this also includes those cases which do not belong to the long established and consolidated democracies. The case of South Africa indicates that even if governments obviously followed a path where democratic principles had been violated during the pandemic, this has not been prolonged after the crisis so that the country returned to its pre-pandemic democratic standards. This leads, second, to another and so far neglected aspect: The potential trajectory that erodes democratic principles and introduces more authoritarian ones, is a conscious and intentional move of the incumbents and their government (and party). Thus, in the case of Hungary, Victor Orbán already had set the path towards an illiberal rule, weakening of horizontal accountability and executive aggrandizement before the pandemic and therefore used the pandemic for further violations of democracy. Hence, in order to capture the ‘real’ impact of the pandemic on democracy, it is important to distinguish between incumbent’s deliberate choice to intensify democratic erosion or autocratization reflecting a long-term project on one hand side, and the temporary decrease of democratic quality during the pandemic which reflects a short-term reaction being reversed at a certain point. Finally, there is another aspect that the contributions of this Special Issue revealed: the preparedness for crisis management. These distinct paths need more comparative and in-depth analysis. In particular, we need to know more about the interaction between the political actors in power and their intentions (democratic erosion and autocratization), the domestic conditions for crisis management and the actor’s choices during crisis situation.

Several studies in this Special Issue point to the issue of public trust. The analyses evince that public trust in government and its crisis management rocketed during the acute phase of the pandemic. This result resonated other studies (Gozgor 2022; Jäckle et al. 2022; Hegewald and Schraff 2022) that show that mitigating measures are more accepted the more people trust in their governments and institutions. These results have to be taken with caution, however, because they only reflect the attitude during the acute phase and not the whole picture including later phases. Thus, the contributions of this Special Issue go beyond a partial view and show that trust did deteriorate in later phases of the crisis. More than that, for those cases where democratic quality decreased, the question remains which long-term effect on public trust this can have.

Indeed, long-term effects remain to be studied. Today it is possible to analyze the short- and mid-term results and consequences of the pandemic from a certain distance in time. Moreover, it might be relevant to distinguish institutional effects (e.g. widering of executive competences) and non-institutional effects such as disruptions of the public discourse, disinformation or manipulation of information, polarization along trust in science etc. Thus, it needs to be explored how far the impact of what the pandemic# triggered in terms of intensifying struggles between democratic and anti-democratic forces will endure.

What concerns the performance of democracies compared to their autocratic competitors, interestingly the balance is more mixed than assumed (see Merkel 2020; Schmidt 2022; Wurster 2022). Democracies in general did not outperform autocracies when it came to containing the pandemic or the Covid-19 mortality. Things are looking better for democracies if one also considers further economic and social consequences of the pandemic. Over the course of the different phases of the pandemic more and more democracies managed to adjust their pandemic response strategies to the existing dilemma between saving life and protecting viability. The critical public debates that have repeatedly motivated the government in democracies to adjust their Covid-19 strategies may also have contributed more adapted development.

From a performance perspective, the structural long-term effects of the pandemic are still open and call for scientific observation. Such a major global event as the pandemic, confronting societies, governments, companies, but also every single citizen with new and intensive experiences, is likely to influence and trigger significant chance processes in every country around the globe. Examples for that might be changes in work organization (home office), in the use of new technologies (digitization, vaccines) or in market behavior (online consumption). Looking at the race between democracies and autocracies it is not only about who will emerge stronger out of the crisis in the long term, but also about who will be able to learn a great deal from this crises for future ones. Ending with a rather optimistic view from the perspective of democracies the pandemic showed that many of them were able to develop a (more) balanced crisis reaction strategy combining lives, rights, and livelihoods (see Holbig 2023) than their autocratic counterpart’s showing a high ability to learn. Nevertheless, and as we have moved from the Covd-19 pandemic in a policrisis situation—consisting of the ongoing climate crisis, the Russian war on Ukraine, and the connected energy crisis—, crisis management and the trade-off between democratic principles and coping with extraordinary threats to state and society will remain a challenge for governments and the public and therefore a topic for political science.
